# Whistle in the bronchus

**DOI:** 10.4103/1817-1737.33702

**Published:** 2007

**Authors:** Parvaiz A. Koul, Abdul Wahid, Tariq A. Bhat, Tajamul Hussain

**Affiliations:** *Department of Internal and Pulmonary Medicine, Sher-i-Kashmir Institute of Medical Sciences, Soura, Srinagar, Kashmir, India*

**Keywords:** Bronchus, foreign body, plastic

## Abstract

An 18-year-old male presented with 4 weeks' history of productive cough and fever that had started 1 day after alleged ingestion of a plastic whistle. Multiple courses of antibiotics had proved ineffective. Crepitation and a localized wheeze on right chest were observed on clinical examination. Chest radiograph showed a right-sided pneumonitis. Bronchoscopy revealed a grayish-black foreign body in the right bronchial tree, which was retrieved and found to be the ‘lost whistle.’ The patient improved dramatically following the procedure.

Aspiration of foreign bodies in tracheo-bronchial tree occurs less frequently in adults than in children.[1] Whereas it is suspected in children with acute or subacute pulmonary symptoms, it is rarely considered in adults unless a clear history of aspiration is evident. The elderly and denture wearers appear to be at greatest risk.[2] Foreign body aspiration (FBA) can be a life-threatening emergency requiring immediate intervention; however, symptoms can also go unnoticed for years with serious sequelae.[3,4] The removal of a foreign body (FB) from the respiratory tract generally leads to a rapid recovery. Some of the materials retrieved from the bronchial tree have been organic materials, metal remnants, bone fragments, vegetables, broncholiths, dental prosthesis, endotontic needle, tracheostomy tube, nails, bean seeds and fruit stones.[2] Most of the foreign bodies are organic in nature, the common ones being nuts and seeds in children and food and bones in adults. Inorganic materials are uncommon; some such materials retrieved have been beads, coins, pills, beverage can tops and caps of pens. Aspiration of plastic foreign bodies is very rare amongst adults. Here we present the case of a young male who had inadvertently aspirated a plastic whistle into his right bronchial tree and was totally oblivious of the event. We are unaware of a similar report in the literature.

## Case Report

An 18-year-old boy presented with a history of acute onset of cough of 4 weeks' duration without any preceding viral symptoms. One day later, the patient had intermittent high-grade fever with intermittent rigors and sweating, and the cough was productive of mucopurulent expectoration. The cough was more intense during the initial days and was subsequently better with cough-suppressant medication. He had received multiple antibiotics for 2 weeks without any relief in the fever or the incessant cough. On routine enquiry, 1 day prior to the onset of his symptoms, he had allegedly ‘ingested’ a plastic whistle while blowing it and was confident of having passed it with his stools even as he did not ever notice it in his feces. Clinical examination revealed an average built febrile young boy with a pulse of 100/min, BP of 110/70 mmHg, respiratory rate of 22/min, with examination of the chest revealing a wheeze and coarse crackles on the right middle and lower anterior chest. Rest of the general physical and systemic examination was unremarkable.

Hemogram revealed Hb of 13 gm%; a TLC of 10.0 × 10^9^ /L with 90% polymorphs, 10% lymphocytes and normal platelets. Serum biochemical parameters were normal. Gram's smear of sputum and routine cultures were negative. Radiograph of the chest showed pneumonitis of right lower zone. In view of the chronological association of the symptoms with the history of ingestion of the foreign body, a flexible fibroptic bronchoscopy was performed. A grayish-black, longitudinally placed foreign body was seen blocking the right-sided B8 and B9 segmental bronchi with hyperemia of the lower lobar bronchial mucosa. It was gradually removed by repeated hooking by forceps through bronchoscope. The retrieved foreign body was found to be the ‘lost whistle’, considerably altered in appearance. The patient was put on antibiotics (Amoxicillin + Clavulinic acid) for 2 weeks. His cough had a dramatic regression, and other symptoms and signs improved gradually over a period of 2 weeks and the chest X ray performed on day 15 was normal.

## Discussion

Foreign body aspiration is more common in children than in adults. The peak incidence of FBA in children is during the second year of life and during the sixth decade in adults.[5] The most common site for aspiration in adults is the right bronchial tree because of its more vertical disposition,[6] and a central location is predominant in children. The most frequent symptom of FBA is called ‘penetration syndrome,’ defined as a sudden onset of choking and intractable cough with or without vomiting. The retention of aspirated foreign body (AFB) in tracheo-bronchial tree may also produce recurrent pneumonia, bronchiectasis lung abscess, atelectasis, post-obstructive hyperinflation; and occasionally a misdiagnosis of asthma, COPD or lung cancer is made in adults who have aspirated an FB. Tissue response to a foreign body varies according to the composition of the FB and any associated bacterial infection. Vegetable fragments such as nuts and grains cause greater acute inflammation than pieces of metal, plastic or bone. The relative inert nature of the plastic material of the whistle was the likely reason that was responsible for the relatively quick response of the patient upon removal of the foreign body, implying a milder tissue inflammation. Delay in diagnosis ranges from hours to years and is considerably shorter in children than in adults,[5] possibly due to parental attention. Our patient had 1 month delay in the diagnosis because of the belief that he had passed the ‘ingested’ whistle with feces, as well as the nonvisualization of the foreign body on the radiograph of the chest. Chest X ray usually suggests the site of foreign body, but a non-radio-opaque foreign body may be aided by inspiration-expiration films, which demonstrate air trapping distal to obstructed segment.[7]

Bronchoscopy is usually necessary to establish the diagnosis and attempt removal of foreign body.[8] Rigid bronchoscopes are usually used, but flexible bronchoscopes are increasingly being used and only few patients need to be sent for thoraco-surgical treatment. Better instruments of visualization have been reported to lead to reduced complication rates.[5] Virtual bronchoscopy also can aid in the diagnosis of a foreign body, which can be the prelude to the final retrieval of the foreign body.

Our case strongly illustrates considering the diagnosis of FBA in an adult with acute/subacute pulmonary symptoms and emphasizes that the diagnostic work-up should also include a search for a foreign body that was forgotten.
